# Progress towards Every Newborn Action Plan (ENAP) implementation in Iran: obstacles and bottlenecks

**DOI:** 10.1186/s12884-021-03800-x

**Published:** 2021-05-17

**Authors:** Fariba Mirbaha-Hashemi, Batool Tayefi, Zahra Rampisheh, Arash Tehrani-Banihashemi, Mozhdeh Ramezani, Narjes Khalili, Omid Pournik, Rahim Taghizadeh-Asl, Abbas Habibelahi, Mohammad Heidarzadeh, Maziar Moradi-Lakeh

**Affiliations:** 1 Preventive Medicine and Public Health Research Center, Iran University of Medical Sciences, Tehran, Iran; 2 Preventive Medicine and Public Health Research Center, Department of Community and Family Medicine, School of Medicine, Iran University of Medical Sciences, Tehran, Iran; 3 Statistics and Information Technology Management, School of Medicine, Iran University of Medical Sciences, Tehran, Iran; 4 World Health Organization, WHO Representative’s office, Tehran, Iran; 5 Neonatal Health Office, Ministry of Health and Medical Education, Tehran, Iran; 6 Department of Neonatology, Tabriz University of Medical Sciences, Tabriz, Iran; 7 Preventive Medicine and Public Health Research Center, Psychosocial Health Research Institute, Department of Community and Family Medicine, School of Medicine, Iran University of Medical Sciences, Hemmat Freeway, Next to Milad Tower, Tehran, Iran

**Keywords:** Community health, Health equity, Health services development, Health systems, Neonatal health, Qualitative research

## Abstract

**Background:**

Neonatal mortality accounts for more than 47% of deaths among children under five globally but proper care at and around the time of birth could prevent about two-thirds of these deaths. The Every Newborn Action Plan (ENAP) offers a plan and vision to improve and achieve equitable and high-quality care for mothers and newborns. We applied the bottleneck analysis tool offered by ENAP to identify obstacles and bottlenecks hindering the scale-up of newborn care across seven health system building blocks.

**Methods:**

We applied the every newborn bottleneck analysis tool to identify obstacles hindering the scale-up of newborn care across seven health system building blocks. We used qualitative methods to collect data from five medical universities and their corresponding hospitals in three provinces. We also interviewed other national experts, key informants, and stakeholders in neonatal care. In addition, we reviewed and qualitatively analyzed the performance report of neonatal care and services from 16 medical universities around the country.

**Results:**

We identified many challenges and bottlenecks in the scale-up of newborn care in Iran. The major obstacles included but were not limited to the lack of a single leading and governing entity for newborn care, insufficient financial resources for neonatal care services, insufficient number of skilled health professionals, and inadequate patient transfer.

**Conclusions:**

To address identified bottlenecks in neonatal health care in Iran, some of our recommendations were as follows: establishing a single national authorizing and leading entity, allocating specific budget to newborn care, matching high-quality neonatal health care providers to the needs of all urban and rural areas, maintaining clear policies on the distribution of NICUs to minimize the need for patient transfer, and using the available and reliable private sector NICU ambulances for safe patient transfer.

**Supplementary Information:**

The online version contains supplementary material available at 10.1186/s12884-021-03800-x.

## Background

The first 28 days after birth, the neonatal period, is the most critical time for a child’s survival. More than 47% of deaths among children under five in 2018 occurred in their neonatal period [1] and 80% of neonatal deaths happened around the time of birth worldwide [2]. An estimated 2.5 million newborns died in the first month of life in 2017 – approximately 7000 every day – mostly in the first week after birth [2]. Almost two-thirds of neonatal deaths could be prevented by suitable care at birth and throughout the postpartum period [3]. The major causes of newborn mortality and evidence-based interventions for reducing the mortality rate are well-known; therefore, a roadmap that illustrates a strategic plan to end preventable newborn deaths is essential in health systems.

The Every Newborn Action Plan **(**ENAP) was launched in 2014 as a global roadmap that provides a strategic plan to achieve equitable and high-quality care for mothers and newborns. It offers a clear vision of how to improve newborn health by 2035. Moreover, this roadmap helps eliminate preventable newborn mortality through global and national plans, evaluation, and accountability frameworks [3, 4]. It is estimated that there are 2.6 million stillborn every year, while most of them are preventable [5]. Overall, it is expected that around 3 million lives would be saved each year if ENAP’s evidence-based solutions and actions are implemented [6]. The Ministry of Health and Medical Education (MoHME) in Iran, started to integrate ENAP into the national neonatal health programs in 2016. Since then, all startegic goals and dimensions of ENAP have been integrated with a special focus on “reducing inequties”, “improving quality” and “counting everynewborn through measurement, programme-tracking and accountability”.

ENAP presents evidence-based solutions to formulate a framework to reach specific milestones, including 10 or less newborn deaths per 1000 live births, and ensuring that no newborn is left behind by 2035 [7]. The survival of newborns, especially those who are small and sick depends on the health system response. Thus neonatal deaths can be considered as sensitive indicators of the quality of newborn care in a health system [8]. To strengthen newborn health components in existing health sector plans and strategies that relate to reproductive, maternal and child health, strong national leadership is required to reach the ENAP goals.

In Iran, according to the World Health Organization (WHO) statistics, the neonatal mortality rate was estimated to be 9.6 per 1000 live births in 2016 [1]. Although, Iran has already achieved the main targets of ENAP at the national level there are still geographical disparities and inequalities in neonatal mortality, and maternal mortality among different provinces in Iran [9, 10]. In addition, achieving the international targets does not have any conflict with the fact that there are still lots of opportunities to reduce neonatal mortality, and improve maternal, neonatal and children care in our country [11]. The aim of this study was to identify the most important obstacles and bottlenecks in implementing ENAP in Iran to help reduce provincial disparities in neonatal and maternal mortality rates and to improve overall maternal and neonatal care. In addition, some recommendations are proposed to overcome these obstacles.

## Methods

### Data collection

In this study, we used qualitative methods to collect information and assess health system bottlenecks in scaling up maternal and newborn care. We used the every newborn bottleneck analysis tool which was developed to assist countries in the identification of these bottlenecks across the seven health system building blocks of “leadership and governance”, “health financing”, “health workforce”, “service delivery”, “essential medical products and technologies”, “health information system”, and “community ownership and partnership.” [12–14]

Identifying the strengths and weaknesses of these building blocks can help determine unmet needs and bottlenecks of neonatal care [15]. Iran has a specific integrated system for “health services” and “education of medical sciences”. Each part of the country, and its population, is covered by one of the public universities of medical sciences. Chancellors of these universities are responsible not only for the research and education of students but also for the health of the population in their catchment area. We purposefully selected 5 (out of 51 at the time of sampling) medical universities and their corresponding hospitals (please see Table 1 in the appendix for details). Our sample of medical universities included different types of universities based on their classification. The classification is a ranking system mainly based on the size of the medical universities. Type 1 medical universities are the largest universities and usually are located in major cities, and type 3 (smallest medical universities) are usually located in smaller cities. Type 2 medical universities are in between. In each of the selected universities and also a private hospital in the capital city, we interviewed the following individuals at their workplaces:

Vice-chancellor (VC) of treatment, VC of public health, deputy director of treatment, director of midwifery services, director of family health, director of neonatal care (if assigned), director of medical devices and commodities, head of departments of neonatology, pediatrics, obstetrics and gynecology, director of nursing services, and also head, supervisor(s) and representatives of healthcare providers (physicians, nurses, midwives) in each of the hospitals. Furthermore, we interviewed national experts and key informants including the head of Iranian Society of Neonatology in Tehran, the director and experts at the national office of neonatal care at MoHME in Tehran, relevant key informants from the United Nations Children’s Fund (UNICEF) and United Nations Population Fund (UNFPA) country offices in Tehran at their workplaces.

The interviews were based on section I of “every newborn bottleneck analysis tool” (Supplement) [8]. The tool facilitates the identification of bottlenecks that hinder the scale-up of facility-based newborn care across seven health system building blocks mentioned above. Participants identified the main bottlenecks and in some areas proposed potential solutions to overcome the bottlenecks under each health system building block.

### Data analysis

We prepared a verbatim transcript from the tape recording of each of the interviews and qualitatively analyzed their content. Informed consent was obtained to record all of the interviews and anonymity was maintained in the transcripts. Each of the transcripts was separately reviewed by two experienced researchers and dissimilarities were discussed and resolved. They were analyzed and coded based on the health system building blocks from the every newborn bottleneck analysis tool. In other words, in this study, we used the seven health system building blocks from the every newborn bottleneck analysis tool as our themes for coding the transcripts of the interviews. Then the identified bottlenecks were categorized under each of the building blocks.

In addition, we reviewed and qualitatively analyzed the performance reports of neonatal care and services from 16 medical universities around the country. These periodic reports included the day-to-day operational challenges the neonatal health unit and care providers encountered in the medical universities. In addition to our interviews in this study, we included these reports as another source to identify challenges and obstacles of neonatal care and services.

## Results

The main identified bottlenecks for scaling-up neonatal care in Iran were as follows:

### Leadership and governance

Participants in this study pointed out many challenges in the leadership and governance of neonatal care in the country (please see Table 3 in the supplements for a summary). The participants (in Kerman, Bushehr, Mazandaran, and provincial reports) suggested that a single entity should be in charge of newborn care to engage all experts and make improvement in newborn care a national priority. It was indicated that many decisions are made by politicians who are focused on their political short-term goals. For example, the decision to have a neonatal intensive care unit (NICU) or a hospital in a small town is sometimes made by a member of parliament (with specific political aims). The participant in Bushehr added, after the political aim is reached or the situation has changed, the project is left unfinished and incomplete because it was not started with proper plans, budget allocation, and situation or need assessments. Many policies and strategies were suggested (in Mazandaran, Kerman, and provincial reports) to be based on current directors’ individual tastes rather than careful situation analysis, immediate needs, and potential consequences.

Developing, disseminating, and enforcing implementation of appropriate policies and guidelines for all levels and sectors of newborn services were argued (in Tehran, Bushehr, and provincial reports) to be major governance bottlenecks.

There were arguments for and against the decentralization of NICUs in the country (in Tehran, Bushehr, provincial reports). One expert in Bushehr argued:

Instead of decentralization of NICUs and spreading the facilities and resources for neonatal care all over the provinces, making the centers of the provinces stronger is more efficient. Other factors such as the distribution of population and distance between the cities in a province should also be considered.

Those who supported decentralized intensive services (in Bushehr and provincial reports), on the other hand, emphasized the need for better access in small towns far from the capital of the provinces.

### Health finance

Most participants perceived financial management as a major bottleneck in neonatal care. Because of a centralized procurement system, expensive equipment and supplies are purchased and assigned to the hospitals. Sometimes hospitals receive equipment that they do not necessarily need but simple instruments that they desperately require are not provided to them (provincial reports). Many of the participants (in Mazandaran, Bushehr, and Tehran) thought that if the budget is given directly to the university hospitals, they could prioritize purchasing equipment that they need.

A participant in Tehran explained,

“In England, for example, neonatologists are paid a certain fixed amount. Their pay is not based on the number of patients they have. Because of this system, only the high risks newborns stay in the NICUs based on specific indicators which are checked daily.”

In Iran, however, the more patients the physicians have, the more they get paid. As a result, the newborns might stay in NICUs longer than they require. He added that other NICU care providers (such as nurses); however, were underpaid for their level of expertise, responsibilities, and workload.

Fee for service payment system had other critics, as well. One participant in Bushehr suggested that it has negative effects on the education of the new generation of health professionals:

The physician payment system was suddenly changed to the fee for service which is problematic. In the old system, the faculty was able to focus on educating the new generation of caregivers and used to hold teaching rounds, for instance. But now, they spend their time going from one hospital to the next to be able to collect fees for their services.

Another major bottleneck was reported in the provincial reports to be the heavy financial debt of university hospitals to pharmaceutical companies. This barrier limits adequate support for special needs of neonatal units in the hospitals. Despite the Coordinating Council in the provinces, insurance companies continue to follow their own guidelines and only have limited coverage for NICU interventions and post-discharge services. Improving the financial process between insurance companies and health care facilities was also suggested in Mazandaran to be required (please see Table 4 in the supplements for a summary).

### Health workforce

Participants in this study identified many challenges in the health workforce (please see Table 5 in the supplements for a summary). Specifically, the number of skilled staff, their distribution, quality of training, and education were described as inadequate. Hospitals in small towns away from the center of provinces suffered from a high turnover of skilled healthcare professionals (reported in Bushehr and provincial reports). The hospitals in the private sector and the capital of provinces offered significantly higher wages and better facilities and thus employed and retained the most eligible workforce (indicated in Bushehr and Tehran). Shortage of specialized workforce such as pediatric surgeon and experts to screen and manage retinopathy of prematurity in public hospitals were also highlighted in Mazandaran, Bushehr, and provincial reports.

In addition, organizational charts were reported (in Bushehr and provincial reports) to be outdated which caused various workforce management challenges. Public hospitals were not authorized to hire a sufficient number of specialized personnel. A participant in Bushehr remarked, “NICUs have progressed well in terms of hardware, but they do not have enough specialized workforce.”

It was argued in Tehran that the guidelines and curriculum for education and training of midwifery and NICU nursing students have been updated but they are not properly implemented and students do not receive sufficient competency training. Most of their training is now theoretical. This leads to the accumulation of an unskilled workforce and low confidence in their abilities, and in the case of midwives, a shift to a preference for obstetrics and gynecologists. Insufficient budget, involvement of university professors, and supportive supervision were also reported (in Tehran and provincial reports) as shortcomings in the training of neonatologists.

### Health service delivery

Many barriers to effective health service delivery were identified such as the poor linkage between health facilities (public-public and public-private) including referral systems, patient transfer, and follow-up programs. Participants reported (in Tehran and provincial reports) that an established referral system was lacking and current transfers were mostly done through personal connections. Significant problems in the safe transfer of newborns were noted (in Bushehr and provincial reports) which included lack of a dedicated expert transfer team, a skilled physician, and proper thermo-regulation and oxygen supply.

Access to quality healthcare was also limited in some areas (as reported in Mazandaran and provincial reports) because of inadequate distribution of NICU beds and equipment. Some hospitals had extra resources such as ventilators that other hospitals needed desperately. Furthermore, obstacles in screening and management of retinopathy of prematurity were reported (in Bushehr and provincial reports) in many hospitals. Limited opportunities for the parents of NICU patients to communicate with their newborns’ physicians were noted (even in a private hospital in the capital city) since physicians visit their patients twice a day, once in the morning and once in the afternoon, and were thus available at the hospital for a very limited time. Moreover, effective post-discharge follow-ups were lacking in public hospitals but some private hospitals offered innovative follow-up systems through social media and the internet (i.e. use of e-health and m-health platforms).

Finally, access of non-Iranians to maternal and neonatal care was noted in provincial reports to be inadequate in some provinces because illegal residents do not have medical insurance and intensive care is not affordable for uninsured people. As a result, a significant number of them suffered from obstetric complications (please see Table 6 in the supplements for a summary of bottlenecks in this building block).

### Essential medical products and technologies

In this study, bottlenecks in the procurement system and supply chain which result in shortage and maldistribution of equipment and medical products were identified as the most challenging in this health system building block. Purchase of capital (more expensive) equipment, in the last 4 years, has been centralized in MOHME. As a result, sometimes a higher number of a type of equipment is provided to the hospitals than is requested but they do not get other devices that they really need (noted in Bushehr and provincial reports). For the less expensive equipment, hospitals are instructed to purchase them from the hospital’s income which can also be problematic because of their limited budget and heavy financial debt. One expert in Tehran noted, “It is hard to say which system, (the new) centralized or (the old) decentralized procurement system, is more inefficient.” He acknowledged the problems with the current centralized system but also mentioned that in the past when the system was decentralized, “sometimes the oldest and least up-to-date neonatologists were invited for procurement consultation and whatever they said was done.”

Moreover, the procurement system was described as ineffective and poorly managed. Some of the imported equipment and devices were claimed to be outdated and substandard. A participant in Tehran explained,

Poor choices are made. For example, equipment made in 2004 was purchased and imported, production of which was already stopped by the company. Because of its outdated technology, the new features could not be added to it so it was practically useless.

He added that end-users and experts have to be consulted about the specification of equipment to be purchased.

Shortage of some inexpensive equipment such as blenders, resuscitation tools, neopuff, and portable echosonography was also reported (in Mazandaran, Bushehr, and provincial reports). The national equipment allocation system was reported to be inefficient and unfair so unofficial connections had to be used to obtain the necessary equipment (noted in Mazandaran). After receiving the equipment, the personnel was not trained properly for the appropriate use of equipment. For instance, some of the options of purchased ventilators were said in Bushehr to be too complicated to be used by the personnel (please see Table 7 in the supplements for a summary of bottlenecks in this building block).

### Health information systems

The health information system was reported to be outdated and incompatible with the current needs of neonatal care. It does not capture all basic and intensive newborn care services and thus documentation of rendered clinical services is incomplete. For instance, sufficient options to record fluid therapy, antibiotic therapy, normal findings of ultrasonography, negative culture, thyroid function test, and details of oxygen therapy are not available, according to the provincial reports. Few personnel, in some hospitals, are in charge of data entry and management, as a result, accurate and timely integration of newborn care activities into the hospital information system (HIS) is limited. In addition, since only few personnel are in charge of the management of the HIS, most of the bottlenecks in this building block came from the provincial reports rather than the individuals we interviewed, who were not mostly involved with this activity.

Inability to generate reports, to be used for reviews and decision-making, was also widely reported in the provincial reports. Data were said to be collected about diseases such as meningitis, jaundice, Retinopathy of Prematurity (ROP), a variety of respiratory problems like pneumothorax and respiratory distress, pulmonary arterial hypertension, neurological problems like asphyxia and seizures, and activities such as resuscitation, but a report about these conditions and their recovery or success rate could not be generated. Moreover, according to the provincial reports, universities did not have access to their hospitals’ data for analysis and reviews. They also reported glitches in the HIS when they needed to link the prenatal information to the maternal and newborns information.

Participants in Mazandaran also reported ambiguity in protocols of perinatal mortality surveillance system and registry. There is a lack of specific linkage between different national mortality registries (*Iranian* Perinatal Mortality Surveillance System (IPMSS), Iranian Maternal and Neonatal Network (IMaN), and National Death *Registry* (NDR). Available data in these databases are incomplete and some of the cases (as noted in Mazandaran and provincial reports) do not have a national identification number for proper identification, tracking, and accurate analysis (please see Table 8 in the supplements for a summary of bottlenecks in this building block).

### Community ownership and partnership

Inadequate public knowledge about vaginal delivery, available maternal and newborn health services, and patient rights (especially among poor and disadvantaged families) were the most common causes of perceived bottlenecks in this area. Some non-Iranian mothers were reported to refrain from seeking health services due to socio-economic or cultural barriers. As a result, as noted in Kerman, out-of-hospital deliveries often occur among this disadvantaged population, and the risk of obstetric complications increases (please see Table 9 in the supplements for a summary of bottlenecks in this building block).

## Discussion

In this study, we identified many challenges and bottlenecks in the provision of newborn care scale-up in Iran. Lack of a single leading entity for newborn care, insufficient financial resources for neonatal care services, insufficient number of skilled staff, and inadequate patient transfer found to be major barriers to optimal neonatal health care.

In the building block of “leadership and governance,” lack of updated guidelines, weak program implementation at lower levels of health care, lack of public-private health care partnership, and ineffective coordination systems were reported in other studies as bottlenecks [8, 16]. In Iran, similar to previous studies, poor central leadership and lack of prioritization of newborn care were identified as the main obstacles [8, 17, 18].

To overcome the identified bottlenecks, the appointment of a national newborn care-specific managing entity with clear accountability for newborn health and survival is necessary. Strong national leadership along with partnerships of organizations central to health care delivery is also imperative. These solutions are among the factors that Darmstadt, et al. named key factors for success in scaling-up newborn health [19]. In addition to strong central national leadership, a focal point and a neonatal care technical committee (involving scientific societies and academics), for each province, can also be helpful (noted in Kerman and Mazandaran).

Close collaboration, partnership, or even integration of maternal and neonatal care organizations is also important. In addition, careful need assessments and well-documented evidence are necessary for developing new national policies and plans. An expert in Tehran argued that the health care system, in general, has to evolve into a system based on consultation, dialogue, and values. Further, neonatal care national guidelines should be adopted as part of the educational curriculum for newborn health care professionals (as suggested in Kerman).

Many participants in this study supported the current decentralized NICU care system. This policy has been supported by the Deputy of Treatment of the MOHME in recent years. However, some experts argued against it and believed that the centralized system is more appropriate. This is an area that requires close monitoring and evaluation because both systems have pros and cons. Furthermore, different provinces might require opposing systems, depending on the factors such as the distribution of population, physical distance to health centers, quality of roads, and access to safe, reliable, and timely patient transfer.

With regard to “health finance,” inadequate funds due to lack of priority and budget line allocation to newborn health, and high out-of-pocket payments were noted as bottlenecks. Out-of-pocket charges for NICU and post-discharge services should be decreased or even eliminated (as suggested in Mazandaran). In addition, a budget line should be allocated to newborn health and interventions, for hospitals to offer standard care and optimal service delivery. The same bottlenecks in neonatal care have been identified in other studies [8, 16–18, 20]. Barriers specific to this study were an under-developed remuneration system, heavy financial debt of hospitals to pharmaceutical companies, inefficient centralized procurement system, and burden of free treatment of the disadvantaged non-Iranian patients for the hospitals (as noted in Bushehr).

The bottlenecks of the “health workforce” included shortage of skilled staff, maldistribution, lack of competency-based training, and lack of retention initiatives especially in small cities and rural areas. The importance of a skilled workforce has been discussed in other studies as well [8, 16–18, 21–27]. Reassessment and development of pre-service and in-service neonatal care education and competency-based training for all levels of care for all involved in maternal and newborn health are necessary. The number and quality of neonatal health care providers’ education and training should also be matched and adjusted to the needs of the health system in the urban and rural areas. In addition, the involvement of university professors and supportive supervision are essential as was suggested by the experts in Tehran and provincial reports.

For “health service delivery,” poor distribution of newborn services especially in the small towns and rural areas, weak referral system and transfer of patients were major bottlenecks identified in this study and other studies as well [8, 18]. Lack of quality monitoring, mentoring and improvement systems were also brought up in previous analyses [8, 16, 18]. Utilization of the private sector NICU ambulances which are reliable and standard for patient transfer was suggested in Bushehr. Involvement of the private sector has been suggested in other studies, as well [8]. If intensive care for newborns is made available in health facilities closest to the ones who need them, the need for patients transfer is also minimized. To improve access and quality of services, utilization of technology and social media, as is already used in the private sector, can also be helpful. In addition, innovative programs such as assigning a “chaperone” nurse for each patient to follow up the patients and ensure high quality of service delivery were also practiced in the private sector. Patient satisfaction monitoring programs were also used and should be mandatory in all hospitals.

In order to improve the quality of care and service delivery, many solutions have been proposed in previous studies. The use of checklists and adherence to standard procedures and protocols such as mandatory use of partograph in the public and private sector, establishing centers of excellence based on practice benchmarks, and institutionalizing integrated supportive supervision and mentorship at all levels of care are among them [8, 16, 17].

When it comes to “essential medical products and technologies,” most of the problems in securing essential newborn products in the country are caused by ineffective procurement system affected by conflicting interests, ineffective and unfair equipment allocation, the high debt of hospitals, and their inability to obtain essential products and commodities on their own. Therefore, solutions consist of establishing effective and transparent procurement systems, streamlining the procurement procedures, establishing an efficient, transparent, and fair equipment allocation system, and empowering hospitals to budget for regular service and repair of their equipment (as proposed in Bushehr). Relevant solutions in other studies on health system bottlenecks include strengthening Logistics Management Information Systems and capacity building of health workers about procurement and supply chain management systems [8, 16].

For the “health information system” building block, the major identified bottlenecks were outdated HIS, poor documentation of collected data, and lack of linkage between prenatal data to the maternal and newborn information. Ambiguity in protocols of the perinatal mortality surveillance system was also pointed out. To address these barriers, regular updates of the national health management information system to include all neonatal indicators and services are required. A regular capacity-building effort for all healthcare personnel is also essential for accurate data entry and management. It is also crucial for personnel, managers, and statisticians to be able to generate proper and solid reports for routine analysis and interpretation to incorporate feedback and for further programmatic actions.

Proposed solutions in other studies that may prove to be useful for Iran include, incorporating neonatal intervention-specific indicators into HIS (such as the use of partograph and provision of kangaroo mother care), engaging the private sector in providing data for key indicators, institutionalizing data quality assurance, and strengthening e-health system using mobile phones, which are already ubiquitous around the country, to follow up postnatal care [8, 17, 18].

Barriers for “community ownership and partnership” included inadequate public knowledge about newborn care issues, entitlement, and services which have been brought up in previous studies as well [8, 16–18]. Delay in care-seeking due to socio-economic and cultural barriers was also important in this analysis and previous ones [8]. Recommendations in this study consist of communicating messages about newborn health and increasing public knowledge, empowering women (especially the impoverished and poor immigrants) through health education and information sharing in the communities, involving non-governmental organizations (NGOs) in supportive and educational programs, and mobilizing charities to help build and establish birth centers and provide essential equipment for neonatal care such as incubators and phototherapy (as suggested in Bushehr and provincial reports). In addition to these points, discussion in community forums, engaging communities and leaders in town hall meetings or focus group discussions, involving communities in planning and implementing newborn health initiatives, male involvement, and use of social media are useful suggestions, proposed in previous studies [8, 16–18].

We included different types of public universities (and their corresponding hospitals), as well as a private hospital in our study. We also interviewed neonatal experts in the MoHME and key informants in the UN agencies related to family and childcare. We met these experts at their workplace so that it would be the most convenient for them. However, like any other qualitative study, our data may not be representative of all bottlenecks of neonatal health care across the country and this is a limitation of our study.

Despite all of these challenges, neonatal care in Iran has achievements and strengths as well. Some of these capacities are listed in Table 2 in the appendix. Most notably, Iran has already reached the international target of 10 or less newborn deaths per 1000 live births and had an estimated Neonatal Mortality Rate (NMR) of 9.6 in 2016 [1, 7]. Other improvements include: appropriating Baby-Friendly Hospitals, forming a newborn resuscitation team in teaching hospitals, and educating parents on newborn care through midwives and educational material. Some of these scale-up plans are carried out only as pilot programs and only in a few specific hospitals and as a result, regional disparities in neonatal health might still occur [9, 10].

## Conclusion

In this study, key stakeholders in the provision of newborn care services provided important insight into factors that are important and hinder the quality and coverage of neonatal care in Iran. Neonatal care is the most sensitive test of universal health coverage and quality of care [28, 29]. Goals for child survival and reduction of neonatal mortality cannot be reached without an increased focus on neonatal outcomes [30–33]. It is imperative that all inter-sectorial players come together to overcome the bottlenecks and ensure quality and equitable newborn care for all. Strong leadership is essential and proper policy, guidelines, and standards are needed to formulate, implement, maintain and monitor at all levels of maternal and newborn services in public and private sectors [34–40]. In addition, a social approach is required to involve all sectors and stakeholders in improving maternal and neonatal health.

Based on the results of this study, specific recommendations to address bottlenecks of neonatal health care in Iran include: establishing a single authorizing body and leadership entity at the Ministry of Health level and the affiliated medical universities, establishing a multi-disciplinary technical committee to make decisions on technical issues based on evidence and assessments, allocating specific budget to newborn care, maintaining clear policies on decentralization of NICUs (location and number of beds) to minimize the need for patient transfer, utilizing the available and reliable private sector NICU ambulances for safe patient transfer, analyzing root causes of regional disparity in neonatal outcomes and indicators, strengthening vital registration systems, and allocating neonatal care resources in a more efficient and equitable way to minimize the regional disparities. National neonatal health plans should continually be assessed using quality data and be adjusted to meet the needs of the country. Health workforce planning at all levels is also imperative to provide the right care for every newborn baby and leave no newborn behind.

### Supplementary Information

**Additional file 1.**

**Additional file 2.**

**Additional file 3.**

**Additional file 4.**

**Additional file 5.**

**Additional file 6.**

**Additional file 7.**

## Data Availability

We conducted interviews in this study and prepared a verbatim transcript from a tape recording of each of the interviews. Informed consent was obtained to record all of the interviews and anonymity was maintained in the prepared transcripts from the interviews. These transcripts are available from the corresponding author on reasonable request.

## Appendix

**Table 1:** Visited health care facilities during the field observations

Province	Mazandaran	Kerman	Kerman	Bushehr	Sistan and Balouchestan
**University of Medical Sciences**	Mazandaran University of Medical Sciences	Kerman University of Medical Sciences	Rafsanjan University of Medical Sciences	Bushehr University of Medical Sciences	Zahedan University of Medical Sciences
**Type**	Two	One	Two	Three	Two
**Referral Hospital (City)**	Bou-Ali Sina Teaching Hospital (Sari), Imam Khomeini Teaching Hospital (Sari)	Afzalipour Teaching Hospital (Kerman), Payambar-e Azam Social Security Hospital (Kerman)	_	Shohaday-e-Khalij-e Fars Teaching Hospital (Bushehr)	_
**District Hospital (City)**	Imam Hossein Hospital (Neka), Shohda Hospital (Behshahr)	Shahid Ganji Hospital (Sirjan)	Hazrat-e Ali-ebn-e-Abi-Taleb Teaching Hospital (Rafsanjan)	Imam-Khomeini Hospital (Kangan), ShahidGanji Hospital (Borazjan)	_
**Delivery Facility center (District)**	_	Haaj-Zangiabadi Delivery Facility Center (Zangiabad)	_	_	_
**Health Center (District)**	_	Zaniabad Rural Health Center (Zangiabad)	_	_	_
**Heath House (District)**	Shakta Health House)Kolijan Rostaq)	_	_	_	_

**Table 2:** Strengths and capacities of neonatal health care in Iran

• Achieving international targets on neonatal mortality at the national level	
• Considerable decrease in neonatal mortality rate despite the increasing trend of preterm babies	
• Designating and increasing coverage of Baby-Friendly Hospitals	
• Improving physical space and amenities of hospitals through Labor and Delivery Room (LDR)	
• Increasing number and access to NICU beds	
• Increasing the presence of resident pediatricians or neonatologists at hospitals and during high-risk childbirths	
• Training and monitoring of general neonatal care, such as well-baby care, skin-to-skin contact, breastfeeding, and Kangaroo Mother Care (KMC).	
• Training and monitoring of specialized care such as neonatal resuscitation, Acute Care of At-Risk Newborns Program (ACORN), Peripherally Inserted Central Catheter (PICC), and neonatal respiratory care	
• Monitoring hospitals on services such as well-baby program, and high-risk newborn care and sending feedback to the hospitals	
• Organizing a newborn resuscitation team in teaching hospitals	
• Providing various neonatal care educational booklets to healthcare providers	
• Providing various educational pamphlets on newborn care to parents and families	
• Preparation of pathways and protocols for inter-hospital transfer of newborns and referrals by each university of medical sciences • Establishing periodic neonatal mortality clinical rounds in each hospital	
• Implementation of Nursery Assessment and Certification Program (NNACP) as a pilot program	
• Providing mothers’ education for neonatal care during pregnancy by certified midwives	
• Providing breastfeeding counseling to mothers to promote breastfeeding in some hospitals	
• Launching the first breast milk bank (Alzahra Hospital, Tabriz, 2016)	
• Re-orienting charities and utilizing donations to buy required equipment such as phototherapy and incubators in some hospitals	
• Launching a pilot project on home care newborn program	
• The screening program for hearing loss in newborns	

## Abbreviations

ENAP: Every Newborn Action Plan
WHO: World Health Organization
MoHME: Ministry of Health and Medical Education
UNICEF: United Nations Children’s Fund
UNFPA: United Nations Population Fund
NICU: Neonatal intensive care unit
HIS: Hospital information system
ROP: Retinopathy of prematurity
IPMSS: *Iranian* Perinatal Mortality Surveillance System
IMaN: Iranian Maternal and Neonatal Network
NDR: National Death *Registry*
NGOs: Non-governmental organizations
NMR: Neonatal Mortality Rate
