# Supplementary material for: Progress towards Every Newborn Action Plan (ENAP) implementation in Iran: obstacles and bottlenecks
Source: BMC Pregnancy Childbirth. 2021 May 17;21:379. doi: 10.1186/s12884-021-03800-x (PMC8127274; doi:10.1186/s12884-021-03800-x)
Supplement: Supplementary file 1 — Additional file 1. [file 12884_2021_3800_MOESM1_ESM.docx]

| ***Table 3. Bottlenecks in scaling-up neonatal care in Iran, in the health system building block of “Leadership and governance”*** | | |
| --- | --- | --- |
| ***Category*** | ***Identified bottlenecks*** | |
| Structure of the neonatal care system | | - Lack of strong national neonatal policy-making entity - Disintegration in the system of governance and leadership across the country - Lack of cohesion between the strategies and operational plans of the two MoHME deputies which are in charge of “Public Health” and “Treatment affairs” - Lack of partnership among private hospitals, public university hospitals, and Social Security Organization hospitals - The weak private sector in some provinces |
| Political support and coordination | | - Lack of national priorities for improvement of neonatal care - Insufficient sensitivity to the specific needs of neonates - Weak attention to preventive and prenatal care compared to the treatment care - Delayed access and poor compliance to national guidelines in private hospitals |
| Process of decision making | | - Insufficient focus on situation analysis as the base of national policy and strategies - Lack of engagement of all stakeholders in new-born care - Making decisions based on individual tastes rather than on systematic strategies - Lack of transfer of lessons learned from one group of management to the next - Decisions are made for political gains and not informed by evidence and immediate needs - Sudden change of policies without considering the consequences - Focus of politicians on short-term gains instead of long-term goals |
