# Supplementary material for: Progress towards Every Newborn Action Plan (ENAP) implementation in Iran: obstacles and bottlenecks
Source: BMC Pregnancy Childbirth. 2021 May 17;21:379. doi: 10.1186/s12884-021-03800-x (PMC8127274; doi:10.1186/s12884-021-03800-x)
Supplement: Supplementary file 2 — Additional file 2. [file 12884_2021_3800_MOESM2_ESM.docx]

| ***Table 4. Bottlenecks in scaling-up neonatal care in Iran, in the health system building block of “Health finance”*** | | |
| --- | --- | --- |
| ***Category*** | ***Identified bottlenecks*** | |
| Funding and budget allocation | | - Insufficient funds for equipping and organizing all neonatal units across the provinces as well as education and in-service training - Inconsistency between the physical expansion of some of the NICUs and distribution of equipment such as ventilators - Inappropriate centralized system for purchasing expensive equipment - Inconsistency between the budget and resources for neonatal care and functions of the Deputies of Treatment Affairs and Public Health of the MOHME |
| Payment system | | - Under-developed payment system which pays physicians based on the number of newborns they have in the NICUs - Disproportionate compensation of the NICU staff to their workload and level of expertise |
| Hospitals' financial problems | | - Heavy financial debt of hospitals and universities to pharmaceutical companies limit adequate support for special needs of neonatal units in the hospitals. - Burden of treatment expenses of the disadvantaged non-Iranian patients for the hospitals |
| Insurance services | | - Lack of proper regulation of insurance companies and their guidelines - Narrow scope of health insurance coverage of NICU interventions and post-discharge services |
