# Supplementary material for: Progress towards Every Newborn Action Plan (ENAP) implementation in Iran: obstacles and bottlenecks
Source: BMC Pregnancy Childbirth. 2021 May 17;21:379. doi: 10.1186/s12884-021-03800-x (PMC8127274; doi:10.1186/s12884-021-03800-x)
Supplement: Supplementary file 3 — Additional file 3. [file 12884_2021_3800_MOESM3_ESM.docx]

| ***Table 5. Bottlenecks in scaling-up neonatal care in Iran, in the health system building block of “Health workforce”*** | | |
| --- | --- | --- |
| ***Category*** | ***Identified bottlenecks*** | |
| Human resources | | - Shortage of nursing staff, neonatologists, and pediatricians outside the capital of the provinces - Inappropriate distribution of skilled staff between and within provinces - High turnover of doctors and other staff, especially in remote and far-off hospitals - Under-utilization of neonatal transport ambulances due to shortage of skilled manpower |
| Organizational charts | | - Lack of resident physicians at many hospitals, especially in non-academic hospitals - Lack of a dedicated position in charge of neonatal health care in university teaching hospitals and each of the health centers - Lack of authorization to hire enough specialized personnel in public hospitals - Outdated organizational charts cause the majority of the manpower problems |
| Training and education | | - Inadequate training for physicians to deliver appropriate neonatal services - Disregarding updated curriculum and guidelines in education and training - Insufficient practical training for midwifery and NICU nursing education - Suboptimal training of neonatologists and insufficient involvement of university professors in their training |
| Compensation issues | | - Employing and retaining the most eligible manpower in the private sector and capital of provinces because of higher wages, better life chances, and facilities - Long delays in compensation which affects the motivation of skilled personnel |
