# Supplementary material for: Progress towards Every Newborn Action Plan (ENAP) implementation in Iran: obstacles and bottlenecks
Source: BMC Pregnancy Childbirth. 2021 May 17;21:379. doi: 10.1186/s12884-021-03800-x (PMC8127274; doi:10.1186/s12884-021-03800-x)
Supplement: Supplementary file 4 — Additional file 4. [file 12884_2021_3800_MOESM4_ESM.docx]

| ***Table 6. Bottlenecks in scaling-up neonatal care in Iran, in the health system building block of “Health service delivery”*** | | |
| --- | --- | --- |
| ***Category*** | ***Identified bottlenecks*** | |
| Referral system and patient transfer | | - Inappropriate and non-systematic neonatal referral system - Lack of a dedicated skilled transfer team and lack of a proper process of inter-hospital transfer and admission - Problems in the safe transfer of newborns, such as thermoregulation, oxygen supply, and presence of a skilled physician in the ambulance |
| Access to high-quality services | | - Maldistribution of NICU beds, ventilators, and other equipment (unused resources in some hospital and shortage in others) - Inappropriate communication of parents of NICU patients with the physicians - Inadequate access to maternal or neonatal care for non-Iranians in some provinces |
