# Supplementary material for: Progress towards Every Newborn Action Plan (ENAP) implementation in Iran: obstacles and bottlenecks
Source: BMC Pregnancy Childbirth. 2021 May 17;21:379. doi: 10.1186/s12884-021-03800-x (PMC8127274; doi:10.1186/s12884-021-03800-x)
Supplement: Supplementary file 5 — Additional file 5. [file 12884_2021_3800_MOESM5_ESM.docx]

| ***Table 7. Bottlenecks in scaling-up neonatal care in Iran, in the health system building block of “Essential medical products and technologies”*** | | |
| --- | --- | --- |
| ***Category*** | ***Identified bottlenecks*** | |
| Essential medical products and equipment | | - Maldistribution of equipment, physical space, and facilities for NICUs between and within provinces - Lack of resources is one of the reasons for the death of newborns in lots of investigations - Vaginal delivery has been increased following the health transformation plan which in turn has elevated the need for products such as Whole Body Cooling due to the increase of asphyxia |
| Procurement process and supply chain | | - Inadequate attention to expert opinions regarding the purchase and distribution of equipment - Inefficient centralized purchasing system of capital (more expensive) equipment - Inefficient centralized purchasing system has replaced the inadequate decentralized system - Major concerns about importing outdated and substandard equipment and devices because of financial interests of a few individuals - Importance of unofficial connections in the allocation of equipment because of lack of a robust allocation system - Susceptibility of the purchasing system to fraud |
