# Supplementary material for: Progress towards Every Newborn Action Plan (ENAP) implementation in Iran: obstacles and bottlenecks
Source: BMC Pregnancy Childbirth. 2021 May 17;21:379. doi: 10.1186/s12884-021-03800-x (PMC8127274; doi:10.1186/s12884-021-03800-x)
Supplement: Supplementary file 6 — Additional file 6. [file 12884_2021_3800_MOESM6_ESM.docx]

| ***Table 8. Bottlenecks in scaling-up neonatal care in Iran, in the health system building block of “Health information systems”*** | | |
| --- | --- | --- |
| ***Category*** | ***Identified bottlenecks*** | |
| Documentation of patients' information in the information system | | - The outdated electronic information system is not compatible with the current needs. - Duplicates are not flagged in the system. - Inadequate documentation of services provided in NICUs due to the outdated information system - Delay in documentation because of low speed of the internet which causes "error" in the information system - Insufficient number of skilled personnel for data entry in the information system |
| Data linkage in the information system | | - Glitches in the system when linking the prenatal information to the maternal and newborns information   Flaws in the system to link the mother's information to the neonatal system |
| Generation of reports | | - Impossible to generate a report about some conditions and procedures even when the data is available in the system. - Impossible to obtain a report for each hospital from the neonatal information system - Lack of access to the university hospital data from the university headquarters |
| The registration systems | | - Ambiguity in protocols of perinatal mortality surveillance system - Because of multiple registration systems in the country, registration has to be done in all of those systems. - Lack of specific linkage between different databases (IPMSS, IMaN, and NDR) |
| Social inequality | | - Delivery or operating room data of non-Iranian mothers, without a national card, cannot be linked with the newborn's information system. |
