# Supplementary material for: Progress towards Every Newborn Action Plan (ENAP) implementation in Iran: obstacles and bottlenecks
Source: BMC Pregnancy Childbirth. 2021 May 17;21:379. doi: 10.1186/s12884-021-03800-x (PMC8127274; doi:10.1186/s12884-021-03800-x)
Supplement: Supplementary file 7 — Additional file 7. [file 12884_2021_3800_MOESM7_ESM.docx]

| ***Table 9. Bottlenecks in scaling-up neonatal care in Iran, in the health system building block of “Community ownership and partnership”*** | |
| --- | --- |
| ***Category*** | ***Identified bottlenecks*** |
| Community awareness | - Inadequate public knowledge and information about vaginal delivery - Lack of awareness among poor and disadvantaged people who do not use health services about their rights, entitlements, and availability of maternal and newborn health services |
| Socio-cultural barriers | - Lack of care-seeking due to socio-economic or cultural barriers especially among non-Iranian mothers - Many of the out-of-hospital deliveries occur among disadvantaged non-Iranian mothers |
